# Downregulation of Calcineurin Gene Is Associated with Glucantime^®^ Resiatance in *Leishmania infantum*

**Published:** 2013

**Authors:** Mohammad Bagher KHADEM ERFAN, Mehdi MOHEBALI, Elham KAZEMI-RAD, Homa HAJJARAN, GholamHossein EDRISSIAN, Setareh MAMISHI, Mojtaba SAFFARi, Reza RAOOFIAN, Mansour HEIDARI

**Affiliations:** 1 Department of Medical Parasitology and Mycology, School of Public Health, Tehran University of Medical Sciences, Tehran, Iran; 2 Center for Research of Endemic Parasites of Iran (CREPI), Tehran University of Medical Sciences, Tehran, Iran; 3 Pediatric Infectious Diseases Research Center, Tehran University of Medical Sciences, Tehran, Iran; 4 Department of Medical Genetics, Tehran University of Medical Sciences, Tehran, Iran; 5 Stem Cell Preparation Unit, Farabi Eye Hospital, Tehran University of Medical Sciences, Tehran, Iran

**Keywords:** *Leishmania infantum*, Calcineurin gene, Antimonial resistance, cDNA-AFLP, Real-time RT-PCR

## Abstract

**Background:**

Pentavalent antimonials are the first line drugs for the treatment of leishmaniasis. Unresponsiveness of *Leishmania* spp. to antimonial drugs is a serious problem in some endemic areas. Investigations on molecular mechanisms involved in drug resistance are essential for monitoring and managing of the disease. Cal-cineurin is an essential protein phosphatase for number of signal transduction pathways in eukaryotic cells and it has a mediated role in apoptosis. This study aimed to determine of biomarker(s) in Glucantime^®^ resiatance strain of *L. infan-tum*.

**Methods:**

We used cDNA amplified fragment length polymorphism (cDNA-AFLP) and real time-RT PCR assays to compare gene expression profiles at the mRNA levels in resistant and susceptible *L*. *infantum* field isolates.

**Results:**

The cDNA-AFLP results showed downlegulation of calcineurin in resis-tant isolate in comparison with susceptible one. Significant downregulation of cal-cineurin (0.42 fold) (*P*<0.05) was found in resistant isolate compared to susceptible one by Real time-RT PCR.

**Conclusion:**

This is the first report of calcineurin implication in Glucantime^®^ drug resistance of field (natural) isolate of *L. infantum*. Downregulation of calcineurin could protect parasites from antimonial-induced apoptosis.

## Introduction

Several species of *Leishmania* are causative agents of a wide spectrum leishmaniasis which ranging from self-healing skin lesions to dangerous visceral forms (1). Global prevalence of leishmaniasis accounts for 12-15 million people and incidence rate of the disease is estimated 2 million new cases annually (2). Despite importance of the disease and serious attempts of researchers, no desirable vaccine is yet available against the disease. Rapid detection and appropriate treatment in most clinical forms of leishmania-sis have essential role in control of the disease (3–5).

Pentavalent antimonials sbV, such as Glucantime^®^ have been used clinically as the first line drug against leishmaniasis since seven decades (6). Although these drugs have been administrated long time worldwide, some biochemical aspects of sbV metabolism in *Leishmania* are not uncovered (3). The sbV is a prodrug and kills *Leishmania* by sbIII which is an activated form of sbV. The exact site of this activation and involved mechanisms in this process are still unknown (7).

Unfortunately, in recent years the efficacy of antimony therapy has been challenged by occurrence of drug resistance. The major problem in treatment of leishmaniasis in some regions such as India is emerging of sbV-resistant parasites (8). Unresponsiveness to Gluca-ntime^®^ has been also reported from Iran (9).

Various mechanisms are involved in drug resistance of *Leishmania* such as drug entry, drug metabolism, drug transport, programmed cell death (10). For instance, Aquaglyceroporine (AQP1) is a plasma membrane protein of *Leishmania* and involves in entry of activated form of drug sbIII to parasite (11). Transfection and knock out experiments of AQP1 have shown that decrease of AQP1 led to resistance (11, 12). TDR1 and lmACR2 of parasite have been detected which involved in sbV reduction (13, 14). It is well documented that MRPA is one of the most important gene in drug transport and sequestration (12, 15). In addition, some studies illustrated the involvement of Heat Shock Proteins (HSPs) in resistance by modulating some phase of apoptosis pathway (10). Furthermore, it has been suggested that the expression of wild-type calcineurin could be a key element in the processes of apoptosis. In the eukaryotic cells such as cardiac and nerve cells the biological function of this protein is dependent to calcium/calmodulin (16, 17).

In spite of the fact that several techniques such as RT-PCR, proteomics and microarray are currently employed for identification of molecular mechanism that are involved in antimonial resistance of *Leishmania*, some aspects of this issue require further investigations by other approaches (18, 19). cDNA-AFLP as a method allows researchers to monitor trans-criptional changes associated to development and modification in cellular functions. In contrast to some techniques such as GeneChip, microarray which are relied on specific probs, cDNA-AFLP does not need any prior knowledge of gene sequences (20, 21).

In this study we aimed to utilize cDNA-AFLP for identification of potential biomarkers in antimonial drug resistance in *Leishmania*.

## Materials and Methods

### Isolation of parasites

Two natural *Leishmania* spp. isolated from visceral leishmaniasis patients by bone marrow aspirations. One of them was clinically resistant to Glucantime^®^ but another one was sensitive to the drug. Resistant case has been treated three periods with systemic administration of Glucantime^®^ but did not lead to cure. Susceptible case after bone marrow aspiration has been treated with Glucantime^®^ and led to cure. These isolates were confirmed as *L. infantum* by ITS1PCR-RFLP method (22). Parasites were cultured in RPMI 1640 medium (Gibco/BRL) supplemented with 10% fetal bovine and incubated at 25°C.

Ethical Committee of the School of Public Health, Tehran University of Medical Sciences reviewed and approved the study protocol.

### Amastigote drug susceptibility assay

Drug susceptibly of amastigotes of *L. infantum* to Glucantime^®^ was determined by cultivation of the parasites in the J774A.1 monocyte-macrophage mouse line. Briefly, macrophages (5 X 10^4^ macrophages/well) were cultured in RPMI1640 with 10% FBS in eight chamber LabTek tissue-culture slides and incubated at 37 ^°^C for 24 h. In order to infect macrophages by stationary phase promastigotes, 5 X 10^5^promastigotes/well were added to macrophages then incubated at 37^°^C for 4 h. Subsequently; cells were incubated for 72 h with serial dilutions of Glucantime^®^. Pentavalent antimony concentrations for sensitive isolate used 2, 4, 6, 8, 10 and 12 µg/ml and for resistant isolate were 35, 40, 45, 50, 55, 60, 65 and 70 µg/ml (the doses were used based on our previous screening test).Fresh drug was added to slides for an additional 72 h. After staining by Giemsa, based on counting the amastigotes in 100 randomly chosen macrophages, the IC50 is defined as the effective dose of Glucantime^®^ that decreases the survival of *L. infantum* by 50%. Experience was performed triplicate and IC50 values were determined by linear regression analysis.

### RNA extraction

Total RNA was extracted using Tripure kit according to the manufactur's protocol (Roch, Mannheim, Germany) with minimal modifications. Briefly, 1 x10^8^ promastigotes were packed by centrifugation. Subsequently, the pellets were lysed in 1ml Tripure reagent and followed by adding 200µl choloroform. After centrifugation, the aqueous phase was collected and precipitated by adding of 500 µl isopropanol. Then, pellets were treated with 75% ethanol and air-dried. Precipitated RNA was dissolved in RNase free water. The quantity and quality of RNA was examined using nanodrop (ND-1000, Thermo-Scientific Fisher, US) and gel electrophoresis, respectively. The extracted RNA was treated with DNase1 to eliminate any genomic contamination (Qiagen, Germany).

### cDNA AFLP

cDNA AFLP was conducted as described previously (23). Briefly, single strand cDNA (sscDNA) was synthesized with 10µg of total RNA, 10 mM of dNTP (Fermentas, Burlington, Canada), 20pmol/µl OligodT (Fermentas, Burlington, Canada), 20pmol/µl random hexamer (Fermentas, Burlington, Canada) and incubated at 65°C for 10 min following by addition of 200 U RevertAid premium Reverse Transcriptase (Fermentas, Burlington, Canada) 20 U Ribolock RNase inhibitor (Fermentas, Burlington, Canada) and 4µL of 5X reverse transcriptase buffer containing Tris–HCl (PH 8.3) (Fermentas, Burlington, Canada) then incubated at 25°C for 10 min following by 50°C for 60 min. The integrity of cDNA was checked with glyceraldehyde 3-phosphate dehydrogenase (GAPDH) gene primers as housekeeping (Table 1). The PCR condition was: (5min at 94°C; 30 cycles of 30s at 94°C; 30s at 55°C; 45s at 72°C and 7 min at 72°C).Double strand cDNA (dscDNA) was generated using 1.5 U *Ribonuclease* H (Roche, Mannheim, Germany), 40U *DNA Polymerase* I (Fermentas, Burlington, Canada), 10 mM of each dNTP and incubation at 16 °C for 2 h, followed by purification with High Pure PCR Cleanup Micro Kit (Roche, Mannheim, Germany). Double restriction digestion was performed with 5U *Mbo* I and 5U *EcoR* I *(*Fermentas, Burlington, Canada) on 5 µg purified dscDNA at 37°C for 2 h.

**Table 1:** Primer and adaptors sequences used in cDNA-AFLP and Real-Time RT-PCR

NAME	SEQUENCES	PRIMERS
**AD ECOR1**	5′-ACCGACGTCGACTATCCATGAAG -3′	Adaptor
**AD ECOR1**	5′- AATTCTTCATGG -3′	
**AD MBOI**	5′ -CACTATCCAGACTCTCACCGCA -3′	
**AD MBOI**	5′-GATCTGCGGTGA - 3′	
**PR ECOR1**	5′- ACCGACGTCGACTATCCATGAAGAATTC -3′	Pre-amplification
**PRMBOI**	5′- CACTATCCAGACTCTCACCGCAGATC -3	
**S1 ECOR1**	5v- ACCGACGTCGACTATCCATGAAGAATTCC -3′	Sensitive
**S2 ECOR1**	5′- ACCGACGTCGACTATCCATGAAGAATTCG -3′	
**S3 ECOR1**	5′- ACCGACGTCGACTATCCATGAAGAATTCA -3′	
**S4 ECOR1**	5′- ACCGACGTCGACTATCCATGAAGAATTCT -3′	
**S1 MBOI**	5′- CACTATCCAGACTCTCACCGCAGATC -3′	
**S2 MBOI**	5′- CACTATCCAGACTCTCACCGCAGATC -3′	
**S3 MBOI**	5′- CACTATCCAGACTCTCACCGCAGATC -3′	
**S4 MBOI**	5′- CACTATCCAGACTCTCACCGCAGATC -3′	
**G6PDH F**	5′- ATCAACGACGCACTGCTG-3′	Housekeeping
**G6PDH R**	5′- TTCATCCGCTTCCTTAGGC-3′	
**Calcineurin F**	5′-GTTTTCAGTGGACCCAGGAG-3′	Target Gene
**Calcineurin R**	5′-TGAAACTGTCGTACACCTTGAA-3′	

Digested dscDNA fragments were ligated by AFLP adaptors (Table 1) 8µg AD *EcoR* 1, AD *Mbo* I and 4 µg ad *EcoR* I, ad *Mbo* I. Ligation was conducted in a final volume of 60 µl; it was done in following conditions: 1 min at 55 °C, decreasing to 10 °C over 45 min (i.e. 1°C per 1 min) then 6U T4 DNA Ligase (Roche, Mannheim, Germany) was added to reaction and incubated at 4 °C overnight. Purification was carried out by High Pure PCR Clean up Micro Kit (Roche, Mannheim, Germany) then Pre-amplification was performed with pre-amp primers (Table 1) according to this program: 30 cycles of 94 °C, 30 s, 64 °C, 30 s, and 72 °C, 45 s and final extension at72 °C for 7 min.

Pre-amplified products were subjected to PCR using sensitive primers which containing adaptor sequences plus one nucleotide 3’ (Table 1). Products resulting from sensitive amplification were separated on 10% non-denaturating polyacrylamide gel electrophoresis (PAGE) and stained with silver nitrate

### Isolation, cloning and sequencing

Selected bands were extracted from the PAGE. The eluted DNA was re-amplified by selective (sensitive) primers (Table 1) in 30 cycles with the appropriate selective primers (Table 1) from resultant profile. PCR products were checked on 1.5% agarose gel then cloned in to a pGEM-T Vector System I (Promega, Fitchburg. USA). Recombinant plasmids were sequenced for Identification of cloned transcribed-derived factors (TDFs), using universal primers (T7 promotor and SP6) (Bioneer, Seoul, South Korea). Homology searches were done in non-redundant nucleic and protein databases BLAST.

(http://www.ncbi.nlm.nih.gov/BLAST/).

### Real Time PCR analysis

Real-Time (RT-PCR) was conducted to investigate differences in expression patterns of identified gene(s) between resistant and susceptible isolates. Specific primers were designed by primer 3 version 0.4.0, (http://-frodo.wi.mit.edu/) according to identified target gene (Table 1). cDNA was generated from 1µg of total RNA using QuantiTect^®^ Reverse Transcription(Qiagen, Germany) according to manufacturer's instruction.

This experiment was done in triplicate with 20µl volumes using IQ SYBR green Super mix (Takara, Japan), in an RT-PCR machine (Corbett, RG-3000, Australia).The PCR condition was as follows: activation at 95°C for 30s, amplification at 95°C for 5 s, 60°C for 34 s for 48 cycles. Comparing the cycle thresholds (CTs) of the identified genes with housekeeping gene (GAPDH) the relative value of the expression level of each target gene was analyzed by using the 2 ^−ΔΔCT^ method.

## Results

### Susceptibility of Leishmania infantum amastigotes to Glucantime^®^

The IC50 values of sensitive isolate was 5.33±1.15 µg/ml while this value for resistance isolate was 43.33 ±5.77 µg/ml.

### cDNA AFLP

**Fig. 1:**
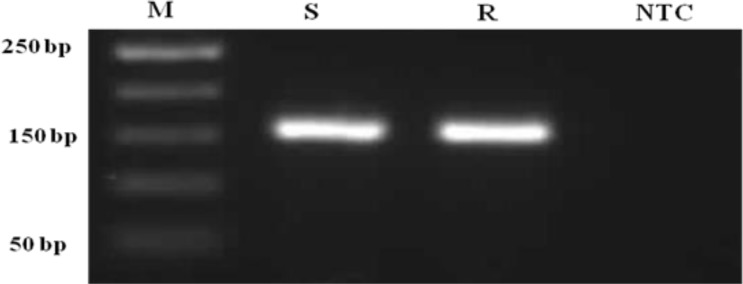
Detection of housekeeping gene, GAPDH, (165bp) by reverse transcriptase-PCR (RT-PCR) on an ethidium bromide stained agarose gel (1.5%). M : 50 bp (base pair) molecular weight marker. S : Sensetive, R : resistant, NTC: No template control

cDNA AFLP approach was conducted to identify differential transcriptomics expression in resistant and suseptible isolates to Glucantime^®^. Total RNA was extracted from samples in logarithmic phase (data not shown). The single strand cDNA (sscDNA) was synthesized as mentioned in materials and methods. Using spesific primers of housekeeping gene (GAPDH), the integrity of cDNA was cheked and confirmed (Fig. 1).

**Fig. 2:**
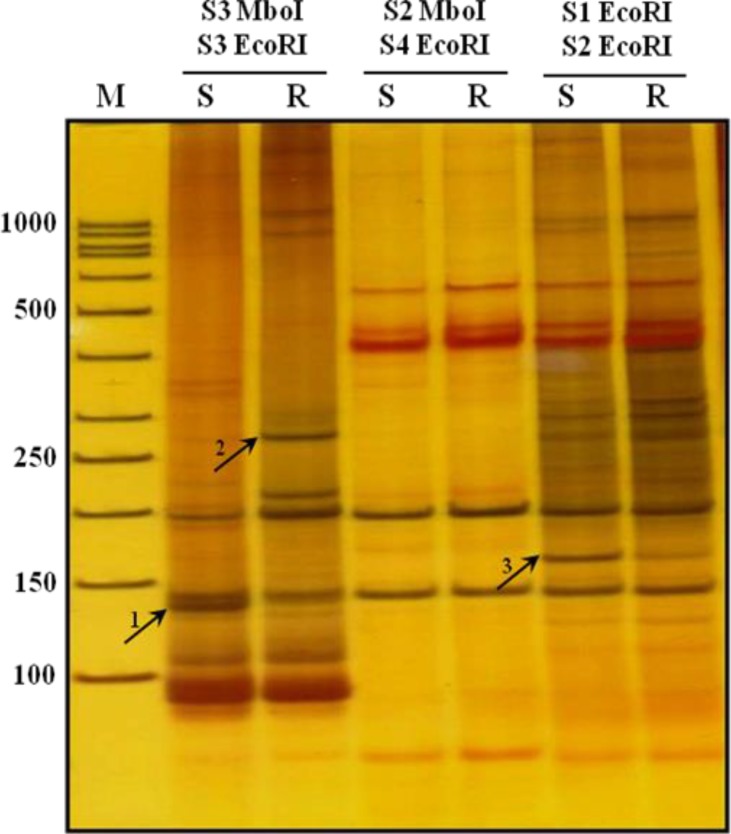
Pattern of TDFs which extracted from cDNA- AFLP PAGE. Sensitive amplification of cDNA AFLP on a PAGE from three different primer combinations: S3 MboI /S3 EcoRI, S2 MboI /S4 EcoRI and S1 EcoRI /S2 EcoRI. The arrows indicate extracted TDFs with cod number: 1, 2, 3 (related genes mentioned in table 2).M; (50 bp) molecular weight marker. S:sensitve. R: Resistant

The cDNA AFLP products from different primer combinations represented on 8% non-denaturating PAGE (Fig. 2).

To detect involved gene(s) in drug resistance mechanisms, different bands containing TDFs were excised from PAGEs and cloned into TA-cloning vector. The recombinant plasmids containing cDNA AFLP products were sequenced. Sequencing results were searched in non-redundant nucleic and protein databases.

In this study, three genes including calcineurin and two unknown genes were identified with different mRNA expression levels in resistant *L. infantum* isolate compared to the sensitive isolate (Table 2).

**Table 2:** Differentially expressed transcription-derived fragments identified by cDNA-AFLP

Code No	Length (bp)	Accession No	Annotation	E-value
**1**	120	XM-001468586.1	Calcineurin	2e-31
**2**	180	XM_001470402.1	hypothetical proteins	1e-12
**3**	260	XM_001470402.1	hypothetical proteins	3e-13

### Validation of cDNA AFLP results using real time RT-PCR

**Fig. 3:**
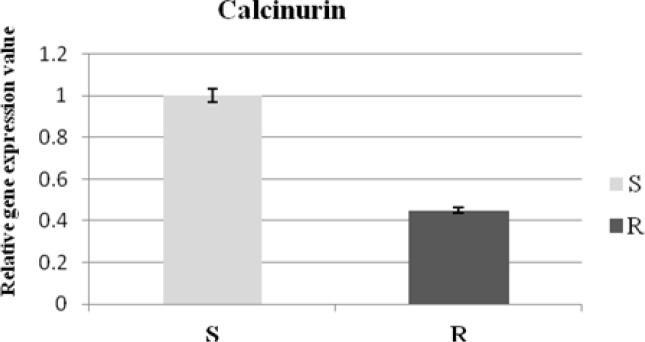
Relative expression pattern of calcinurin gene by real time RT-PCR which was measured in *L. infantum* antimony sensitive compared to resistance. The expression of GAPDH was used to normalize the data. The values are the mean ± SD of at least three different experiments (*P*<0.05)

In order to confirm the cDNA AFLP results, we explored expression of detected gene expressions in samples. Real time RT-PCR discovered the downregulation of calcineurin gene (Fig. 3). Figure 3 shows significant downregulation of calcineurin (0.42 folds) (*P*<0.05) in resistant isolate compare to susceptible.

## Discussion

Since development of effective vaccines against leishmaniasis has not been successful, chemotherapy is the only choice to manage of the disease (3). Emerging of large-scale increase in antimony drugs resistance has been appeared as a serious problem in some endemic regions. To address this problem, several laboratories have undertaken different strategies (18, 19). In the present study, we utilized cDNA-AFLP as a valuable method to discovery gene(s) potentially involved in drug resistance of *L. infantum*. We counted about 1500 TDFs which were differentially expressed in the studied samples.

We found downregulation of calcineurin in Glucantim-resistant isolate compared with susceptible. Calcineurin is a calcium and calmodulin-dependent protein phosphatase and the only protein phosphatase regulated by Ca^2 +^ and involved in diverse cellular activities including, cell survival and apoptosis (24–26).

Several studies reported the roles of this protein in adaptations under different environmental factors, in terms of salt levels and temperature changes (25, 26). For instance, in yeast, adaptation to high salt environment occurs by induction of Pmr2p expression via calcineurin activation (24). Adaptation to environmental temperature changes by calcineurin has been well described in *Cryptococcus neoformans* and *Arabadopsis thaliana* (25, 27, 28). Odom et al. showed its critical role in surviving of *C. neoformans* in serum (25). Calcineurin by interaction with other biomoleculs such as heat shock proteins provides suitable theromtolorance and virulence in *Leishmania major* (29).

In spite of the fact that calcineurin directly or indirectly is implicated in surviving, a line of evidence suggested that under different condition it could play adverse function. For example, calcineurin could trigger apoptosis in different organisms by particular concentration of cytosolic ROS (16, 30). Due to high similarities between mammalian and *Leishmania* calcineurin in terms of function and structure^16^, we suggest that downregulation of calcineurin might have a negative effect on apoptosis in *Leishmania* spp. Consistent with our findings for first time a study by Dhein et al. reported that calcineurin mediates apoptosis in lymphocytes (31). The activation of calcineurin is depended upon cytoplasmic calcium ion concentrations (16). In this regard, a study using cardiac cells revealed that the increasing of intracellular Ca^2 +^ levels led to cellular apoptosis by activation of calcineurin and some transcriptional factors (32). Furthermore, solid evidence supported the implication of elevated intracellular Ca^2 +^ concentrations in *Leishmania* parasite cell death (33–35).

Apoptosis has been also observed and characterized by externalization of phosphatidylserine and nuclear DNA fragmentation in amastigotes of *L. donovani* treatment with antimony compounds (33). Antimony drugs induced the productions of some oxidative agents such as nitric oxide (NO) or hydrogen peroxide (H_2_O_2_) which play a role in leishmanicidal effects of these drugs (36). It is quite understandable that oxidative stress is responsible for elevation of intracellular Ca^2 +^in *Leishmania* and due to this event, apoptosis could be occurred by calcineurin activation.

## Conclusion

For the first time, we identified calcineurin as a drug resistance target gene in *L. infantum*. We suggest that downregulation of this gene might be involved in survival rate of *Leishmania* by inhibiting the apoptosis. However, further studies are needed to define precise biological function of calcineurin in the process of *Leishmania* drug resistance.
